# Overexpression of *SOX3* due to an X chromosome inversion leading to ovotesticular difference in sex development

**DOI:** 10.1186/s13293-025-00822-4

**Published:** 2026-02-12

**Authors:** Carolina Gama Nascimento-Vidoti, Helena Fabbri-Scallet, Mara Sanches Guaragna, Melissa Bittencourt de Wallau, Vanessa Sodré de Souza, Silvia Souza da Costa, Ana Cristina Victorino Krepischi, Juliana Forte Mazzeu, Claudia M. B. Carvalho, Andréa Trevas Maciel-Guerra, Gil Guerra-Júnior, Társis Paiva Vieira

**Affiliations:** 1 Department of Medical Genetics and Genomic Medicine, School of Medical Sciences, Universidade Estadual de Campinas, Campinas, SP Brazil; 2 Laboratory of Human Molecular Genetics, Center of Molecular Biology and Genetic Engineering, Universidade Estadual de Campinas, Campinas, SP Brazil; 3 Faculty of Medicine, University of Brasilia, Brasilia, DF Brazil; 4 Department of Genetics and Evolutionary Biology, Institute of Biosciences, University of São Paulo, São Paulo, SP Brazil; 5 Pacific Northwest Research Institute (PNRI), Seattle, WA USA; 6 Department of Pediatrics, School of Medical Sciences, Universidade Estadual de Campinas, Campinas, SP Brazil

**Keywords:** Structural variants, *SOX3* overexpression, Ovotesticular difference in sex development, Positional effect.

## Abstract

Structural variants (SVs) may increase *SOX3* expression in the gonads and have been observed in individuals with ovotesticular differences in sex development (OT-DSD) and XX testicular differences in sex development (T-DSD). Most of the SVs found in OT-DSD individuals are whole-gene duplications, and to date, only one SV affecting *SOX3* expression by a positional effect has been described. We report an individual raised as a female with *SRY-*negative OT-DSD. Karyotype analysis showed a pericentric inversion in one of the X chromosomes − 46,X, inv(X)(p22;q27). The breakpoints and fusion were mapped using optical genome mapping (OGM) and short-read whole genome sequencing. One of the breakpoints was mapped on Xq27.1 (genomic position chrX:140,420,874 – GRCh38), 82 kb downstream of the *SOX3* gene. This breakpoint was predicted to interrupt a topological associate domain (TAD) affecting 24 enhancer-promoter interactions of *SOX3*. RNA sequencing (RNA-seq) of a formalin-fixed paraffin-embedded (FFPE) sample of the gonads confirmed increased *SOX3* expression. The present study is the first to analyze gene expression in gonadal tissues from an OT-DSD individual, and the first reporting an inversion-based mechanism leading to XX OT-DSD. Additionally, an X-inactivation assay on DNA extracted from the gonads revealed random inactivation. These findings support the hypothesis that inappropriate *SOX3* expression may result from the positional effects of SVs, leading to OT-DSD in 46,XX individuals.

**Supplementary Information:**

The online version contains supplementary material available at 10.1186/s13293-025-00822-4.

## Abstract (plain language summary)

Structural variants are large alterations in DNA (greater than 50 base pairs) that modify chromosome structure. These alterations can create breakpoints that lead to chromosomal reorganization. Additionally, they may alter the expression of genes located within or near the affected region, due to changes in the genomic position of these genes. When such alterations occur in XX individuals and involve the *SOX3* gene or nearby regions, they can lead to overexpression of *SOX3*. This may result in changes in sexual development, leading to conditions known as ovotesticular differences in sex development (OT-DSD) and XX testicular differences in sex development (T-DSD). In the present study, we investigated an XX individual with OT-DSD who presented with an inversion on one of the X chromosomes, initially detected by chromosomal analysis. Using more advanced techniques, we precisely identified the disrupted region and found that one of the breakpoints was located near the *SOX3* gene. Expression analysis demonstrated overexpression of *SOX3*, which correlates with the clinical features observed in this individual.

**Supplementary Information:**

The online version contains supplementary material available at 10.1186/s13293-025-00822-4.

## Highlights

Abnormal expression of *SOX3* and *SOX10* during key developmental stages may induce *SOX9* activation and initiate testis differentiation.

Positional effects resulting from structural variants may contribute to OT-DSD development in 46,XX SRY-negative patients.

The inappropriate expression of the *SOX3* gene observed in patients with OT-DSD may be explained by structural variants involving or located near the *SOX3* locus.

Prediction of topologically associated domains (TADs) at one of the breakpoints revealed disruption of the *SOX3* gene enhancer, and the altered expression was confirmed by RNA-seq.

**Supplementary Information:**

The online version contains supplementary material available at 10.1186/s13293-025-00822-4.

## Introduction

Ovotesticular difference in sex development (OT-DSD) is a rare congenital condition characterized by atypical gonadal sex development. It refers to a situation in which ovarian and testicular tissues are found in the same individual, either in separate gonads or in the same gonad, called an ovotestis. OT-DSD has an estimated incidence of less than 1 in 20,000 individuals, representing less than 3%–10% of all DSD cases. The majority of patients with OT-DSD have a 46,XX karyotype, others may have chimerism (46,XX/46,XY) or mosaicism (for instance, 46,XX/47,XXY), and less commonly, with a 46,XY karyotype [1]. A few patients may present with structural chromosome aberrations [2, 3].

Testicular differentiation is initiated by the expression of the *SRY* gene, which upregulates the *SOX9* and *NR5A1/SF1* genes [4]. In individuals with 46,XX OT-DSD lacking *SRY*, insufficient expression of pro-ovarian genes, or gain-of-function alterations in pro-testicular genes or in their regulatory regions can cause the phenotype [5, 6]. Duplications encompassing the *SOX9* gene or its regulatory regions have been identified in OT-DSD patients who lack *SRY*. Additionally, genomic rearrangements affecting other genes from the *SOX* family, including *SOX3* and *SOX10*, have also been described in OT-DSD, suggesting that abnormal expression of these genes at critical stages of development may activate *SOX9* and initiate testis development [2, 7, 8].

A study by Sutton et al. (2011) showed that increased ectopic *Sox3* expression in undifferentiated gonads can lead to sex reversal in XX transgenic mice, likely by upregulating *Sox9* via a mechanism similar to that of *Sry*. This phenomenon has also been observed in humans, directing the gonads toward male determination [9].

Some patients with 46,XX OT-DSD and duplications of the *SOX3* gene have been reported [2, 10–15]. The regulatory regions that control *SOX3* expression are located at least 500 kb from the gene. Thus, structural variants (SVs) may activate *SOX3* expression in the gonads due to a positional effect [16, 17]. To date, only one individual with OT-DSD and an SV affecting *SOX3* expression by a positional effect has been described [3]. This study describes the clinical and molecular features of a second case.

## Methods

### Cytogenetic analysis

G-banded karyotype was performed on cultured lymphocytes from peripheral blood, with a resolution of approximately 500 bands, according to standard protocols.

### Fluorescent in situ hybridization (FISH)

FISH was performed on lymphocyte cells cultured from peripheral blood, using centromeric probes for the X and Y chromosomes (DXZ1/DYZ3) – (Cytocell - Aquarius^®^), and a locus-specific probe for the Yp11.3 region that includes the *SRY* gene (Vysis - Abbott^®^), with standard protocols. A total of 30 metaphase cells and 200 interphase nuclei were scored for each probe.

### Chromosome microarray analysis (CMA)

This method was performed using genomic DNA sample extracted from peripheral blood using standard protocols. CMA was performed using the CytoScan™ 750 K array from Affymetrix^®^ (Thermo Fisher Scientific Inc. - Life Technologies, Carlsbad, CA, USA) according to the manufacturer’s instructions. Results were analyzed using the Chromosome Analysis Suite (ChAS version 3.3.0.139 (r10838)—Affymetrix^®^) (Thermo Fisher Scientific Inc. - Life Technologies, Carlsbad, CA, USA) with the hg38 human reference genome. Copy number variations (CNVs) were called using a minimum of 50 probes as a reliable indicator for gains and 25 probes for losses. Additionally, regions with at least 500 probes and a size of 1500 kb were considered reliable indicators of homozygous regions.

### Optical genome mapping (OGM)

Peripheral blood samples were collected with EDTA and stored in an ultra-low-temperature freezer (-80 °C) until the OGM procedures. This method was performed at the Uniscience Molecular Laboratory in São Paulo, Brazil, according to the manufacturer’s instructions. Briefly, ultra-high molecular weight (UHMW) DNA was extracted using the Bionano Prep Blood and Cell Culture DNA Isolation^®^ kit (Bionano Genomics, San Diego, CA, USA), employing a nanobind disk to minimize fragmentation, resulting in DNA segments of approximately 150 kb to few megabases (Mb) in size, which are five to ten times longer than the average fragment sizes produced by conventional DNA extraction methods. After extraction, 750 ng of UHMW DNA were fluorescently labeled throughout the genome at a specific sequence motif, specifically the CTTAAG base sequences, using the Bionano Prep Direct Label and Stain (DLS) method (Bionano Genomics, San Diego, CA, USA). The labeled DNA was inserted into a flow cell with parallel nanochannels, where the molecules were linearized and imaged using the Saphyr optical mapping system, generating between 230 and 370 Gb of data per run. The resulting BNX files were analyzed through a bioinformatics pipeline that filtered out molecules smaller than 150 kb. The *de novo* assembly method was performed, and data were aligned to the reference genome (GRCh38) using the Bionano Solve v3.7_03302022_283 RefAligner. Structural variant calling was performed with customized Bionano SV and CNV pipelines. Analysis of SVs, including visual inspection of breakpoints and junctions, was carried out using the Bionano Access v1.7.1.1 software, which enables genome visualization in circos plots and linear maps. The GRCh38 DLE-1 SV mask and GRCh38 CNV masks were used to filter out common SV regions and highly repetitive segments of the genome. This filter contains SV data from 204 individuals from the general population (provided by Bionano Genomics, Inc., San Diego, CA, USA).

### Short-read whole genome sequence (WGS)

WGS was performed to identify additional putative pathogenic variants and fine-map the inversion breakpoints. Sequencing libraries were constructed from 1.0 µg DNA per sample using the Illumina^®^ DNA PCR-Free library preparation kit (Illumina, Inc., San Diego, CA, USA) according to the manufacturer’s protocol. Paired-end sequencing was performed on a NovaSeq 6000 platform, generating > 90 GB of raw data per sample with an average genomic read depth of 30X. Sequence variants were analyzed from the VCF file, while the breakpoint analysis on the X chromosome was performed using the BAM file in Integrative Genomics Viewer (IGV, version 2.17.2) (GRCh38) to identify discordant read pairs and split reads.

### In Silico analysis of TADs disruption

The in silico analysis of topologically associated domains (TADs) disruption by the X chromosome inversion was performed using the TADeus2 website (https://tadeus2.mimuw.edu.pl). This tool evaluates SV breakpoints and assesses their impact on the 3D chromatin architecture, facilitating the interpretation of how alterations in non-coding regions can influence gene expression. Hi-C and Micro-C data of the *SOX3* region were also accessed using the UCSC Genome Browser (https://genome.ucsc.edu/). In addition, ovarian tissue–specific chromatin data were accessed using the ENCODE (https://www.encodeproject.org/experiments/ENCSR181LJD/) and the 3D Genome Browser 2.0 (https://3dgenome.fsm.northwestern.edu/vis?datasets=193), enabling the analysis of the three-dimensional genome organization in a biologically relevant tissue context.

### Transcriptome analysis

Transcriptome analysis was performed on 10 slices (10 μm) of formalin fixed paraffin embedded (FFPE) samples from the patient’s gonads. RNA sequencing libraries were prepared using the TruSeq Stranded Total RNA with Ribo-Zero (Illumina, San Diego, CA, USA) following the manufacturer’s recommendations. Bulk RNA-seq was conducted by *Novogene* using the Illumina NovaSeq PE150 platform, a paired-end sequencing technology with 150-bp read length. Sequencing quality was assessed using FastQC software (version 0.11.2). Raw fastq reads underwent quality control to trim adapter sequences and remove reads with low sequencing quality. Cleaned reads were aligned to the human reference genome (GRCh38 assembly) using STAR (version 2.1.0) [18]. Gene transcript read counts were aggregated into Ensembl Gene IDs for further analysis [19]. Counts were transformed using the Yeo-Johnson method. Transcriptomes of our patient’s gonads were compared with those of individuals of different ages, obtained from the public database E-MTAB-6814 from ArrayExpress [20].

### X chromosome inactivation assay

Genomic DNA was extracted from peripheral blood and from the right gonad. The X chromosome inactivation assay was performed according to Allen et al. [21], with modifications. The methylation status of the highly polymorphic (CAG)n region of the *AR* gene (Xq12) and the (GAAA)n region of *RP2* (Xp11.3) were analyzed. Template DNA was amplified by PCR using primers fluorescently labeled with 6-FAM (6-carboxyfluorescein), flanking the polymorphic regions, and the resulting PCR fragments and signal intensity were determined by capillary electrophoresis using an ABI 3730 DNA Analyzer (Applied Biosystems™). The X chromosome inactivation ratio was calculated as described by [22]. Graphs of X-inactivation were obtained from an analysis conducted using the GeneMarker^®^ program (SoftGenetics, LLC).

## Results

### Patient description

A patient assigned as female was referred for genetic evaluation at three months of age with genital ambiguity. She was the second child of healthy and non-consanguineous parents (father 45 years of age, mother 42 years of age). The mother reported a miscarriage before the proband’s pregnancy, which was uneventful except for hypothyroidism and a urinary tract infection. The patient was born via cesarean section at 39 weeks, with a birth weight of 3.45 kg (+ 0.47 SD), length of 50 cm (+ 0.46 SD), and head circumference of 35 cm (+ 0.95 SD). Hormone evaluation at 1.5 months of age showed normal values for boys and girls in minipuberty of luteinizing hormone (LH) (3.67 mIU/mL) and follicle-stimulating hormone (FSH) (3.09 mUL/mL), and a normal level of testosterone (155 ng/dL) for boys but not for girls. Pelvic ultrasound revealed a normal uterus, but could not identify gonads, while an ultrasound of the inguinal region suggested the presence of a testis on the right. At three months of age, weight was 6.47 kg (+ 0.44 SD), length 61.5 cm (+ 0.28 SD), and head circumference 40 cm (-0.03 SD). She had mild micrognathia and retrognathia, with no other dysmorphisms. Genital examination revealed a 3.1 cm phallus with partial penoscrotal inversion, a single opening between the partially fused, rugated, and pigmented labioscrotal folds, and a palpable gonad in the right inguinal region measuring 0.5 cm in diameter.

At three months, the patient underwent surgical procedures that included exploratory laparoscopy, bilateral gonadal biopsy, cystovaginoscopy, and bilateral inguinal herniorrhaphy. Biopsy of the left gonad revealed the presence of ovarian and testicular parenchyma, compatible with ovotestis, and the right gonad consisted exclusively of seminiferous tubules with Sertoli cells.

Three years later, the right gonad was excised, and histological analysis revealed a small area of ovarian tissue, confirming the presence of an ovotestis. The left gonad, however, was preserved after resection of a small portion of testicular tissue. During the same procedure, female genitoplasty was successfully performed.

The patient had normal psychomotor development and no learning disabilities. At the last evaluation, at 4.2 years, weight was 15.5 kg (-0.47 SD), height 96.9 cm (-1.67 SD), and there were no health issues.

### Karyotyping and fluorescent in situ hybridization (FISH)

**Fig. 1:**
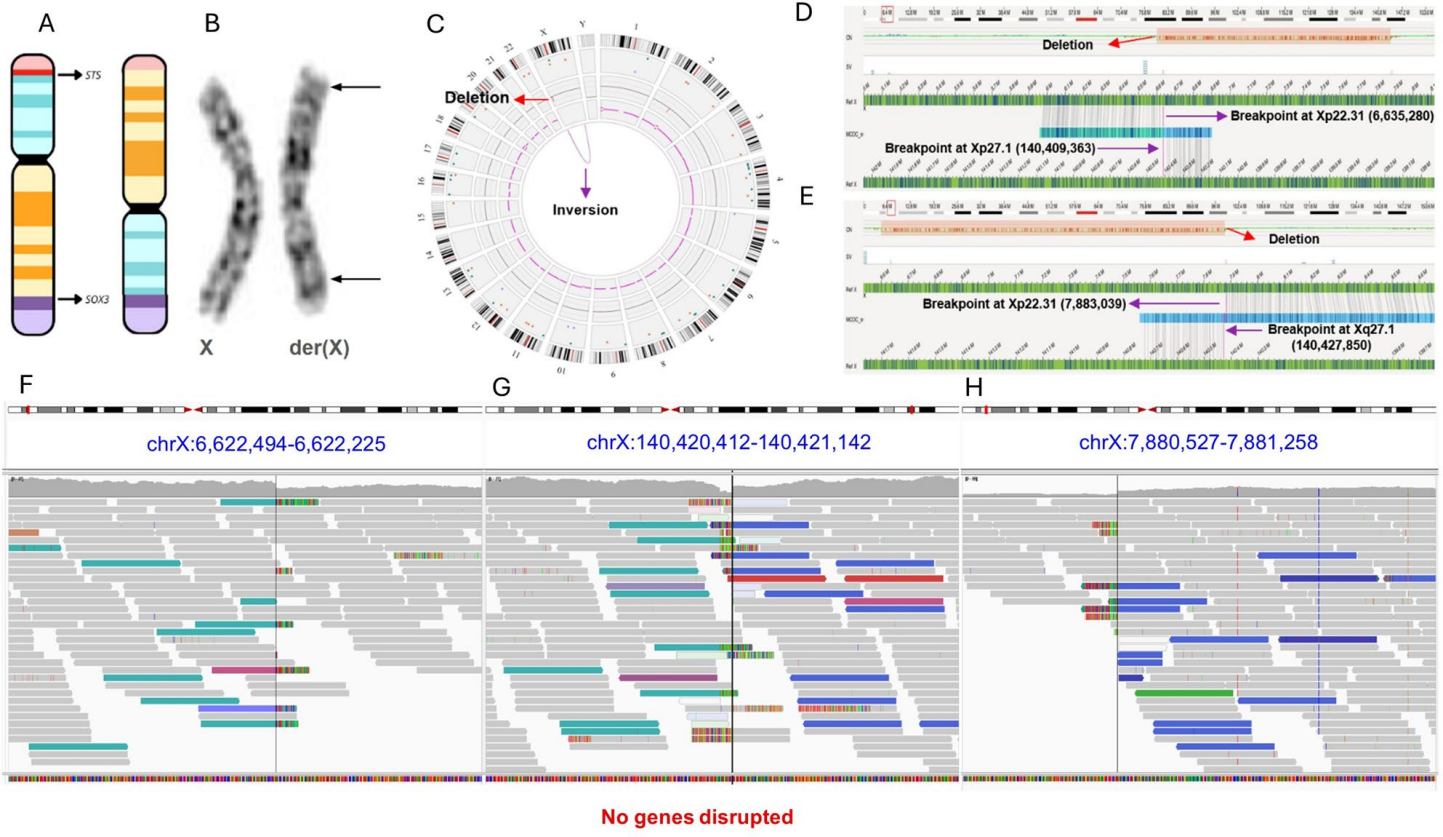
Breakpoint characterization of X chromosome inversion and expression analysis: (A) Ideogram of X chromosome, showing a pericentric inversion and the deletion in the p arm, including STS gene. The SOX3 gene is located near the breakpoint at Xq27.1; (B) Partial G-banded karyotype of X chromosome, showing a pericentric inversion − 46,X, inv(X)(p22.1q26); (C) Circos plot generated by OGM of X chromosome, showing the breakpoints and deletion - ogm[GRCh38] del(X)(p22.31)(6,600,096_7,877,671),inv(X)(p22.31q27.1)(6,600,096 ~ 6,635,280_140,409,363 ~ 140,427,850); (D) Alignment of OGM showing the first breakpoint at Xp22.31 (6,635,280) and Xq27.1 (140,409,363), in purple, before the deletion; (E) Alignment of OGM showing the second breakpoint at Xp22.31 (7,883,039) and Xq27.1 (140,427,850), in purple, after the deletion; (F-H) Images of X chromosome taken from IGV, showing the breakpoints of the inversion: inv(X)(p22.31;q27.1)(6,622,907_140,420,679); inv(X)(p22.31;q27.1)(7,880,769_140,420,874);

G-banding karyotype analysis revealed a pericentric inversion on the X chromosome – 46,X, inv(X)(p22q27) [20] (Figs. 1A and B). Both parents had normal karyotypes.

FISH analysis revealed the presence of two X chromosomes and absence of Y chromosome and *SRY* in all scored cells (data not shown).

### Chromosome microarray analysis (CMA)

CMA revealed a deletion of 1.3 Mb on the short arm of the X chromosome - arr[GRCh38] Xp22.31(6,626,375_7,880,478)x1 (data not shown). This deletion encompasses three protein-coding genes, with only the *STS* gene (OMIM *300747) being haploinsufficient. Hemizygous deletions of this gene are associated with X-linked recessive Ichthyosis (OMIM # 308100), affecting only males. The patient reported herein does not present with ichthyosis, as expected, since most females with heterozygous deletions of *STS* do not exhibit this phenotype. In addition, deletions of *STS* have not been associated with OT-DSD, so this deletion is not causative of the phenotype. No other relevant CNVs were detected on the X chromosome or other genomic regions.

### Optical genome mapping (OGM)

OGM allowed the precise mapping of the X chromosome inversion breakpoints (Fig. 1C). The breakpoints and fusions on the p arm were mapped at Xp22.31, with the first one at the genomic position chrX:6,635,280 and the second at chrX:7,883,039 (GRCh38). The segment between these genomic positions was deleted and was correctly called by OGM, confirming the previous result from CMA. The breakpoint and fusion on the q arm were mapped at Xq27.1, at the genomic position 140,409,363 (GRCh38) – ogm[GRCh38] del(X)(p22.31)(6,600,096_7,877,671),inv(X)(p22.31q27.1)(6,600,096 ~ 6,635,280 _140,409,363 ~ 140,427,850) (Figs. 1D and E). The breakpoints on both arms did not disrupt genes. However, the breakpoint on the q arm was located 82 kb downstream of the *SOX3* gene.

### Short-read whole genome sequence (WGS)

No pathogenic or likely pathogenic sequence variants were found in genes previously associated with OT-DSD. On the other hand, the analysis of discordant read pairs and split reads confirmed and refined the chromosome X breakpoints previously found by CMA and OGM. The 1.3 Mb deletion was mapped between the genomic positions 6,622,907-7,880,774 (GRCh38) at Xp22.31. The X chromosome inversion had two breakpoints on Xp22.31, mapped at genomic positions 6,622,907 and 7,880,774 (GRCh38), and one breakpoint on Xq27, mapped at genomic position 140,420,874 (GRCh38) (Figs. 1F-H). Analysis of the distal Xp breakpoint junction revealed one base pair (bp) of microhomology accompanied by a nearby 11-bp deletion mediated by GCTT microhomology at the first breakpoint junction, between coordinates 6,622,907 and 140,420,874. At the second fusion point, between 7,880,774 and 140,420,874, five bp of microhomology were identified (Supplementary Fig. 1). Formation of a large deletion accompanying this inversion, together with the presence of small microhomology, is consistent with non-homologous end joining (NHEJ) in the SV formation. However, microhomology-mediated break-induced replication (MMBIR) cannot be ruled out. No other relevant SVs were found on the X chromosome or other genomic regions.

### In Silico analysis of TADs disruption

**Fig. 2:**
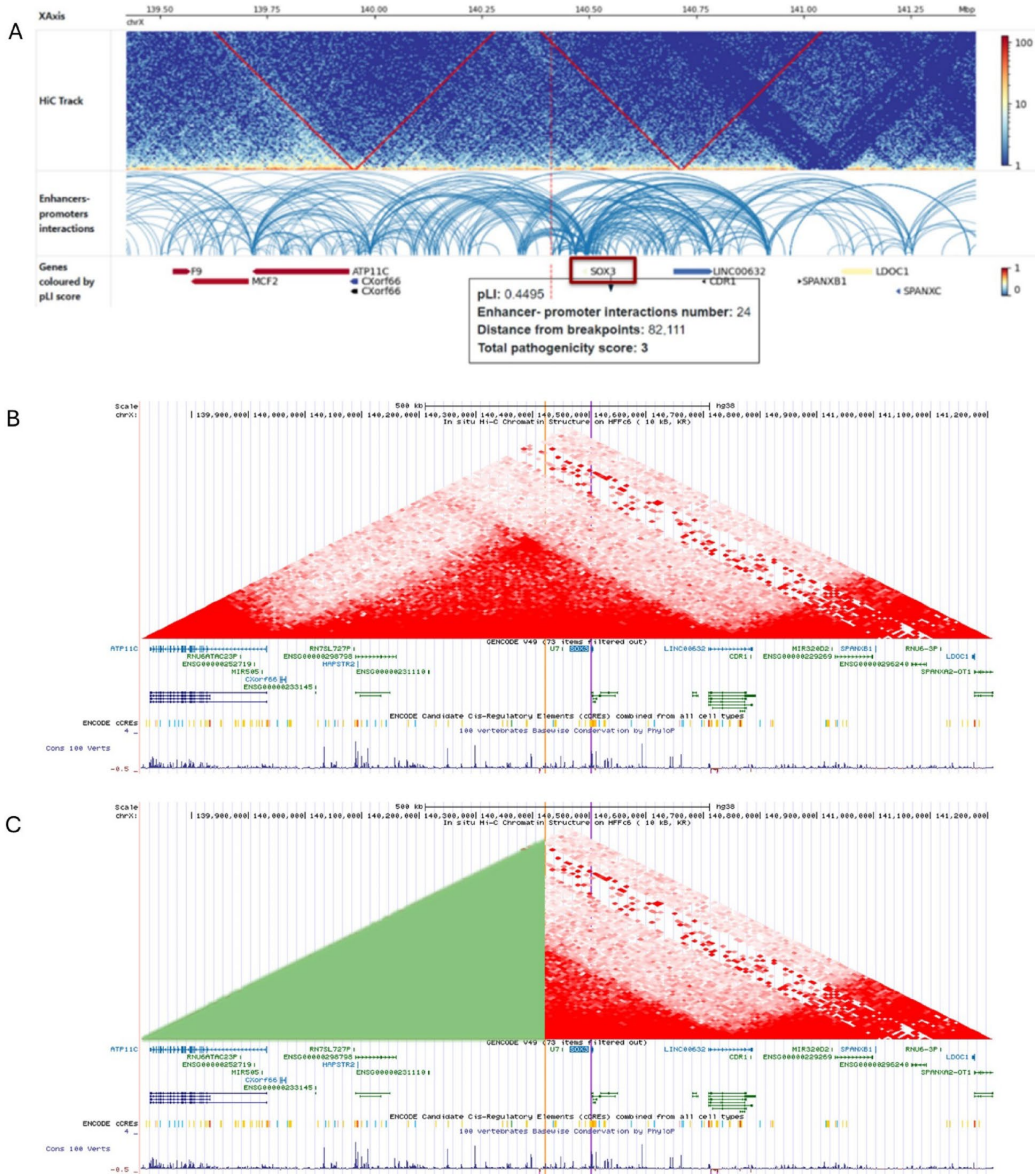
(**A**) Image taken from the TADeus2 website shows positional effect predictions. At Xq27.1, TADs of the *SOX3* were disrupted; (**B**) Visualization of Hi-C maps (10 kb resolution) of the HFFc6 (foreskin fibroblasts) cell line in the UCSC Genome Browser, showing that the breakpoint at Xq27.1 (orange line) and the *SOX3* gene (purple line) are located in the same TAD; (**C**) The inversion (in green) reshuffles this TAD boundary

After accurately identifying the breakpoints, we input these data into the topologically associated domains predictor (TADeus2) to visualize potential disruptions of enhancer-promoter interactions of genes near the breakpoints. This in silico analysis revealed a TAD disruption at the Xq27.1 breakpoint, affecting 24 enhancer-promoter interactions of the *SOX3* gene, resulting in a pathogenic score of 3 (Fig. 2A). The visualization of Hi-C maps (10 kb resolution) of the HFFc6 (foreskin fibroblasts) cell line in the UCSC Genome Browser showed that the breakpoint at Xq27.1 and the *SOX3* gene are located in the same TAD (Fig. 2B), within a continuous domain, and the inversion reshuffles this TAD boundary (Fig. 2C). Therefore, SVs in this region may affect the regulation of this gene.

Data from a HiC assay of ovarian tissue (ENCSR181LJD) in the ENCODE database, showing regions of chromatin interaction and accessibility at Xq27.1, revealed that the breakpoint is located near DNase-hypersensitive sites, a well-established marker of chromatin accessibility. Additionally, the breakpoint is near multiple candidate cis-regulatory elements (cCREs), including enhancers and CTCF binding sites (Supplementary Fig. 2). These elements are defined by integrating chromatin accessibility data and epigenomic marks, indicating regions potentially involved in gene regulation. CTCF binding sites correspond to specific genomic regions where this protein binds and functions as a key organizer of the three-dimensional genome structure. Such sites often act as architectural elements that help delineate regulatory domains and constrain long-range gene interactions. Therefore, the proximity of these elements to the breakpoint suggests a potential impact on local chromatin organization, including TADs. Given the established role of CTCF-associated elements in chromatin architecture, disruption of this region may influence the local topological organization and regulatory landscape of *SOX3*.

The visualization of Hi-C ovarian tissue data in the 3D Genome Browser also showed that the Xq27.1 breakpoint and the *SOX3* gene are within the same TAD, although at very low resolution (Supplementary Fig. 3A). Furthermore, visualization of Hi-C and Micro-C maps (10 kb resolution) of the HFFc6 (foreskin fibroblasts) and H1-hESC (embryonic stem cells) cell lines in the UCSC Genome Browser showed consistency of TAD among different tissues (Supplementary Fig. 3B). Taken together, these data support *SOX3* misexpression as a strong candidate for explaining the patient’s phenotype.

### Transcriptome analysis

**Fig. 3:**
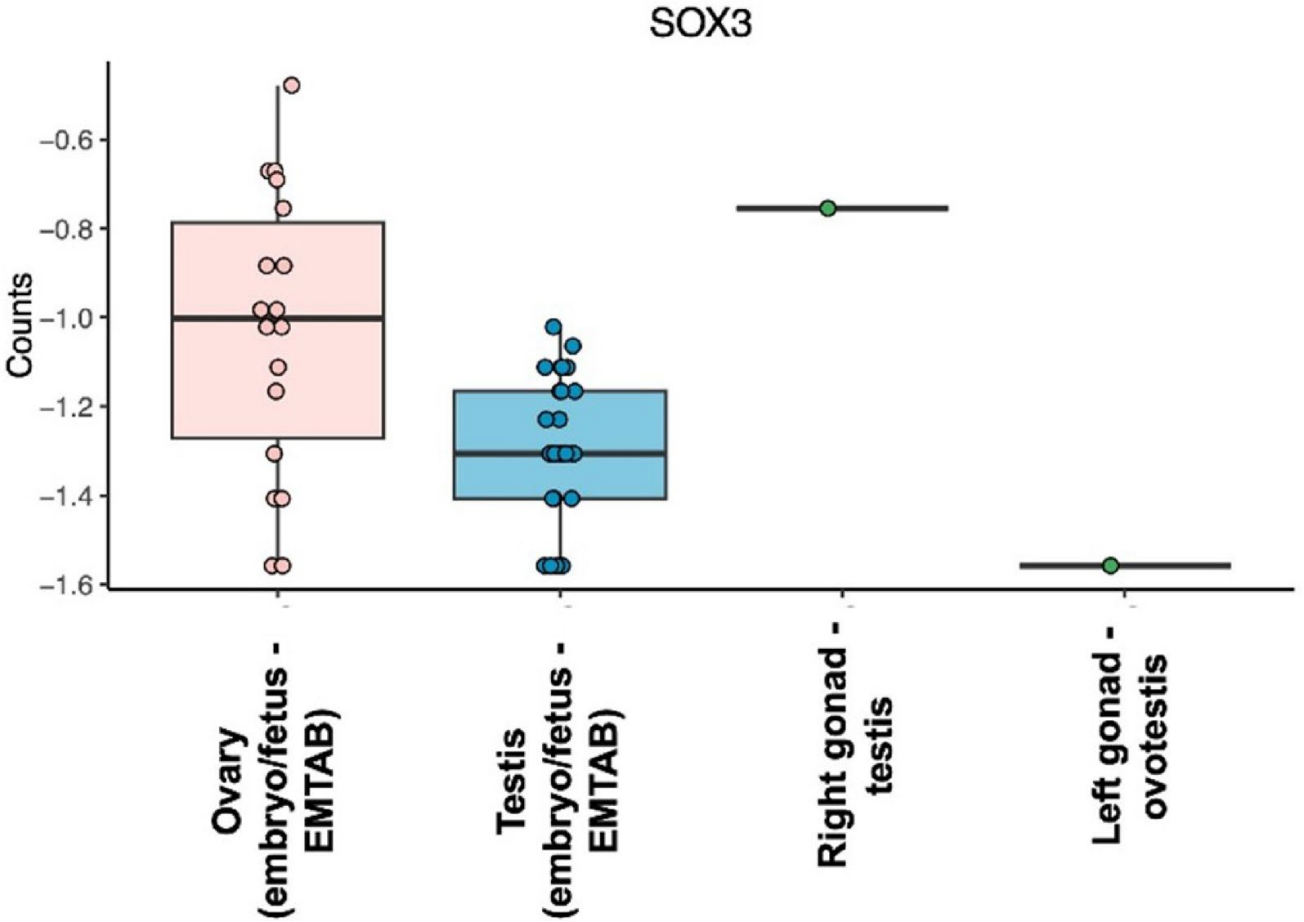
Boxplot showing *SOX3* expression across reference samples and the patient. *SOX3* expression is usually low during the embryonic/fetal stage, as reflected in the controls. In the patient sample, expression was below the median of the embryonic/fetal testis group in the left gonad (ovotestis), whereas the right gonad (testicular tissue) showed higher expression than both the ovary and testis controls

*SOX3* expression was lower in the left gonad (ovotestis), falling below the median of the embryonic/fetal testis group. In contrast, the right gonad (testicular tissue) exhibited increased expression compared to both the ovary and testis controls, confirming *SOX3* overexpression in the patient’s testicular tissue (Fig. 3A).

### X chromosome inactivation assay

The X-inactivation ratios for *AR* and *RP2* in peripheral blood were 77% and 82%, respectively, while DNA from the right gonad showed ratios of 50% and 56%, respectively (Supplementary Fig. 4). These results indicated random X chromosome inactivation in the right gonad, and a relative skewing in blood.

## Discussion

In individuals with XY chromosomes, the *SRY* gene initiates the formation of male gonadal tissue from bipotential gonadal primordia by stimulating a cascade of related genes, including *NR5A1* and *SOX9*, among others. However, in the absence of *SRY*, *SOX9* overexpression in individuals with XX chromosomes is sufficient to induce testis development in the genital ridges [23]. In some individuals lacking *SRY*, duplications or inappropriate expression of *SOX3* has been reported in the literature, as a causes of 46,XX OT-DSD or 46,XX T-DSD [2, 3, 9–12, 14, 15, 24–26].

*SOX3* is a member of the SOX gene family, consisting of a single exon, and located in a highly conserved region of the X chromosome (Xq27.1). Additionally, it encodes a protein closely resembling SRY, with 67% amino acid sequence similarity for the full-length protein and 90% for the HMG (high mobility group) DNA-binding domain. Studies in mice have shown that increased *Sox3* expression in undifferentiated gonads can lead to sex reversal in XX mice. Similarly, in humans, when elevated *SOX3* expression is observed, its protein interacts with NR5A1 to drive the overexpression of *SOX9*, directing gonadal development toward male determination [9].

The individual described in the present study presented with OT-DSD and a pericentric inversion of the X chromosome. Further characterization of the inversion breakpoints using OGM and WGS revealed that one breakpoint, located at Xq27.1, is 82 kb downstream of the *SOX3* gene. According to the prediction of disrupted TADs, 24 interactions involving *SOX3* were disrupted by the inversion breakpoint at Xq27.1, suggesting altered *SOX3* expression due to a positional effect. Additionally, transcriptome analysis of the patient’s gonad confirmed *SOX3* overexpression, and an X-inactivation assay showed random inactivation in the right gonad. Taken together, all these findings strongly support that the OT-DSD in this individual was caused by the X chromosome inversion.

The fine-mapping of the inversion also provided insights into the mechanism of origin. The breakpoint junction alignment supports the formation of the Xp deletion and pericentromeric inversion in the same event, characterizing a complex chromosomal rearrangement (CGR) [27]. These results are consistent with non-homologous end joining (NHEJ) or microhomology-mediated break-induced replication (MMBIR) in SV formation. Inversions accompanied by large CNVs accounted for 17% of those detected by karyotyping, and most were likely generated by replicative repair [28].

The breakpoint at Xq27.1 is located near a palindromic region of approximately 180 bp, which is present only in humans and is genomically unstable, prone to breakage, and represents a critical hotspot for genomic rearrangements. This region is flanked by a long interspersed nuclear element-1 (LINE-1) and long terminal repeat (LTR) sequences [29]. Recurrent rearrangements mediated by the Xq27.1 palindrome have been described and associated with distinct phenotypes, including congenital hypertrichosis, congenital ptosis, Charcot–Marie–Tooth neuropathy (CMTX3), hypoparathyroidism, retinal dystrophy, congenital laryngeal abductor paralysis, Split-Hand/Foot Malformation (SHFM), and other congenital malformation phenotypes [30]. The patient herein described presented with OT-DSD, with no other congenital malformations, and normal development. Although different phenotypes have been associated with rearrangements in this locus, it remains unclear how different phenotypes can result from similar alterations in this region [29, 30].

Most of the individuals with 46,XX OT-DSD or T-DSD and SVs involving *SOX3*, reported in the literature, had duplications encompassing the entire *SOX3* gene (Table 1) [2, 9–12, 14, 15, 24, 26]. In contrast, Sutton et al. (2011) described a patient with T-DSD with a deletion of 343 kb upstream to *SOX3*. The coding sequence of *SOX3* was not affected in this patient, suggesting that altered regulation of this gene was the cause of 46,XX T-DSD. Regarding OT-DSD, only one individual with 46,XX OT-DSD and *SOX3* inappropriate expression due to a positional effect caused by an SV has been described in the literature (Table 1) [3]. Furthermore, only a few studies have performed gene expression analyses of *SOX3* in patients with 46,XX OT-DSD or T-DSD. The present study is the first to analyze gene expression in gonadal tissues from an OT-DSD individual, and the first reporting an inversion-based mechanism leading to XX OT-DSD, supporting that SVs affecting regulatory regions of *SOX3* can cause its overexpression in the gonads.

**Table 1:** Structural variants reported in the literature, involving *SOX3*, in 46,XX *SRY*-negative patients

Gene	Pathogenic mechanisms	Molecular findings	Diagnosis	Genomic positions (GRCh38)	References
***SOX3***	Inappropriate expression	Rearrangement of *SOX3* regulatory sequences	46,XX OT-DSD	inv(X)(p22.31;q27.1)(6,622,907_140,420,679);	This study
			46,XX OT-DSD	ins(X:1)(q27;q25.2q25.3)	Haines et al. [3]
		Duplication of *SOX3*	46,XX T-DSD	Patient A duplication of 6 Mb including *SOX3* gene	Sutton et al., [9]
				Patient C duplication of 123 kb including *SOX3* gene	
			46,XX T-DSD	Xq27.1(140,272,694_140,766,499)x3	Moalem et al., [12]
			46,XX T-DSD	Xq27.1q27.3(140,422,556_146,038,786)x3	Vetro et al., [2]
			46,XX OT-DSD	Xq27.1(140,459,572_140,961,698)x3	Grinspon et al. [13]
			46,XX T-DSD	Xq27.1(140,450,689_140,998,489)x3	Tasic et al., [14]
			46,XX OT-DSD	Xq27.1q27.2(140,417,613_142,689,996)x3	Zhuang et al., [10]
			46,XX OT-DSD	Xq27.1q27.2(140,503,629_141,908,866)x3	Wei et al., [15]
			46,XX T-DSD	Xq27.1(140,445,228_140,719,116)x3	Oroz et al., [24]
			46,XX OT-DSD 46,XX DSD	Xq27.1(140,275,145_141,001,939)x3	de Oliveira et al., [11]
		Deletion located downstream of the *SOX3*	46,XX T-DSD	Xq27.1(139,530,720_140,397,998 )x3	Qin et al., [25]

## Conclusion

In conclusion, we report the second individual with an SV at Xq27.1 resulting in *SOX3* overexpression, and the first one with an inversion leading to XX OT-DSD, which contributes to understanding the distinct mechanisms underlying OT-DSD in individuals with XX chromosomes.

## Supplementary Information

Supplementary Material 1

## Data Availability

The data that support the findings of this study are available from the corresponding author upon reasonable request.
