# Supplementary material for: Overexpression of SOX3 due to an X chromosome inversion leading to ovotesticular difference in sex development
Source: Biol Sex Differ. 2026 Feb 12;17:51. doi: 10.1186/s13293-025-00822-4 (PMC12997796; doi:10.1186/s13293-025-00822-4)
Supplement: Supplementary file 1 — Supplementary Material 1 [file 13293_2025_822_MOESM1_ESM.docx]

chrX:6.622.868-6.622.949


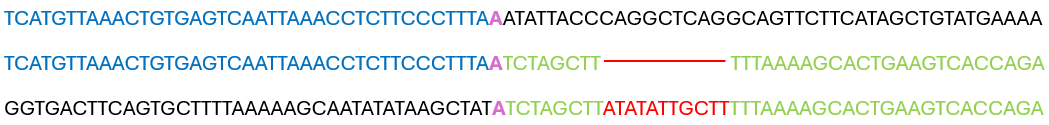


1º breakpoint and fusion point

chrX:140.420.736-140.420.818

A

chrX:140.420.754-140.420.831


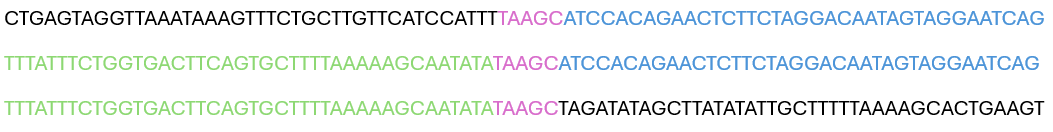


2º breakpoint and fusion point

chrX:7.880.729-7.880.809

B

**Supplementary Figure 1** **–** Representation of the breakpoints and fusion points according to the WGS findings in the IGV analysis software. (A) The sequences in blue represent the alignment of chrX from position 6,622,868 to 6,622,907 (breakpoint is shown in violet). The sequences in green represent the alignment from 140,420,775 to 140,420,818. The region highlighted in red indicates an 11 bp deletion in the patient’s sequence. Therefore, it can be concluded that this first breakpoint contains a microhomology. (B) The sequences in blue represent the alignment of chrX from position 140,420,794 to 140,420,831. The breakpoint is shown in violet, demonstrating the 5 bp microhomology among the three sequences. The sequences in green represent the alignment from 7,880,729 to 7,880,769. Therefore, it can be concluded that this second breakpoint contains a microhomology.


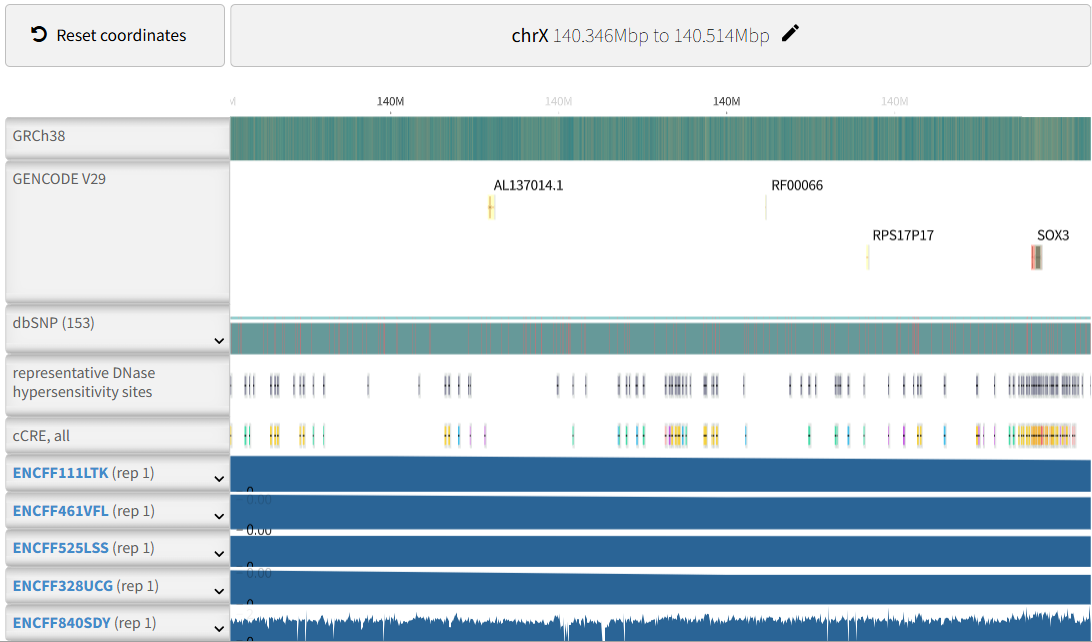

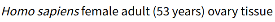


**Supplementary Figure 2 –** Image obtained from ENCODE database, showing regions of chromatin interaction and accessibility at Xq27.1, generated with HiC assay of ovary tissue, suggesting a potentially relevant regulatory context for *SOX3*. Red line represents the approximate breakpoint position. The breakpoint is located within multiple DNase-hypersensitive sites and candidate cis-regulatory elements (cCREs), including enhancers and CTCF binding sites.


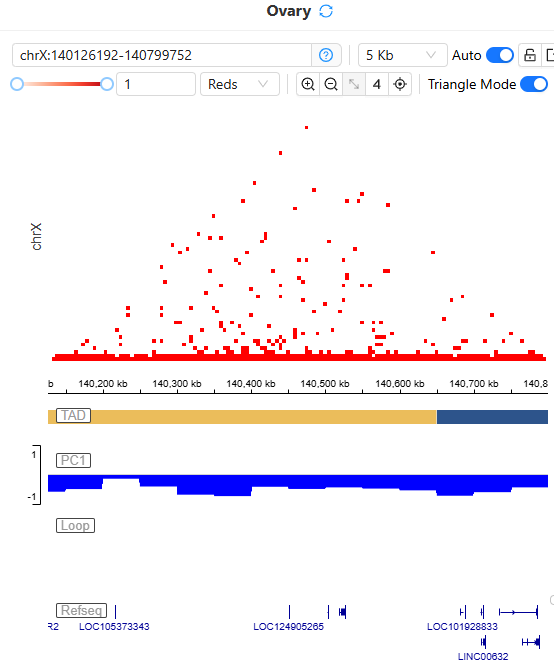


***SOX3***

Breakpoint Xq27.1


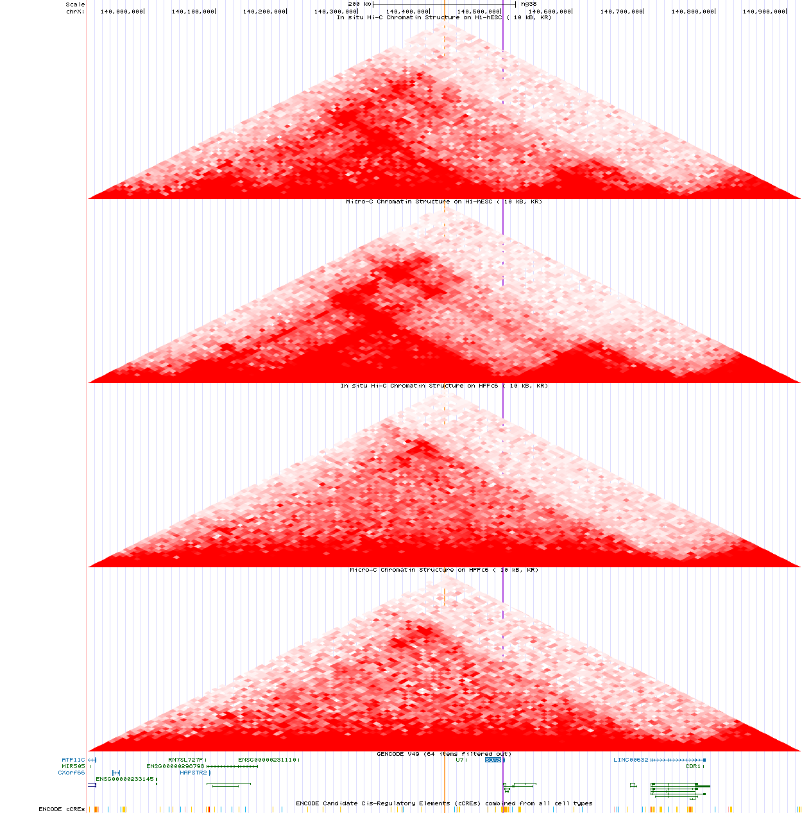


A

B

**Supplementary Figure 3 – (A)** Image obtained from the 3D Genome Browser demonstrating the organization of TADs, with the breakpoint at Xq27.1 highlighted in green and the SOX3 gene in blue, both located within the same topological domain. **(B)** Image of Hi-C and Micro-C maps (10 kb resolution) in the UCSC Genome Browser of H1 hESC cells (H1 human embryonic stem cells) and HFFc6 (foreskin fibroblasts) cell lines, showing consistency of TAD in the *SOX3* region. The orange line is in the Xq27.1 inversion breakpoint, and the purple line is in the *SOX3* gene.


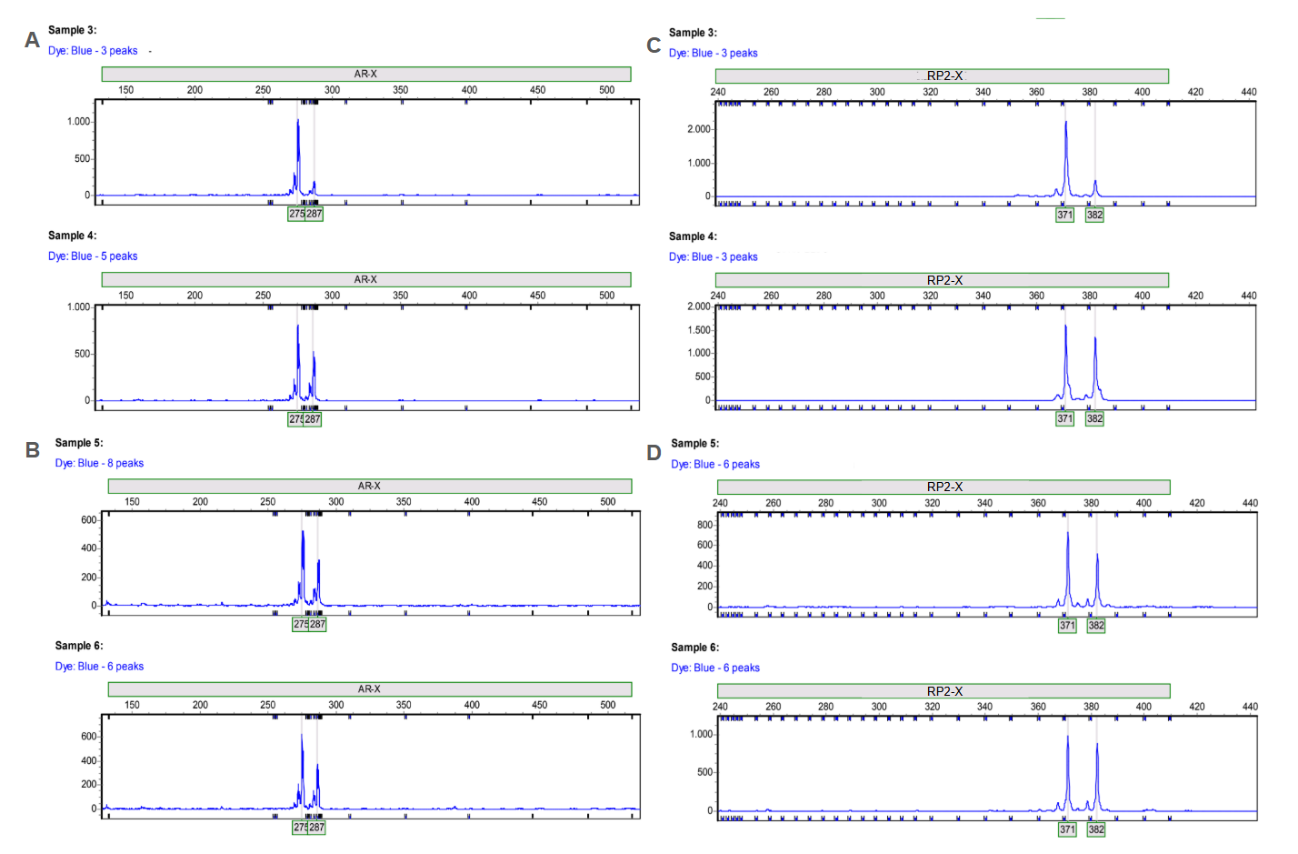
**Supplementary Figure 4** – The graphs of X-inactivation were obtained from an analysis conducted using the GeneMarker program, showing random inactivation. The ordinate and abscissa represent fluorescence intensity and fragment length, respectively. The numbers next to the fluorescence peaks indicate their heights.
(A) The graphs display the peaks identified in the peripheral blood sample, with the first representing the digested sample and the second representing the undigested sample at the *AR* gene locus; the ratio is 77%. (B) The graphs depict the peaks from the patient's right gonad, with the first representing the digested sample and the second representing the undigested sample at the *AR* gene locus; the ratio is 50%. (C) The graphs illustrate the peaks identified in the peripheral blood sample, with the first representing the digested sample and the second representing the undigested sample at the *RP2* gene locus; the ratio is 82%. (D) The graphs show the peaks from the patient's right gonad, with the first representing the digested sample and the second representing the undigested sample at the *RP2* gene locus; the ratio is 56%.
